# Predicting Childhood Anaemia in Nigeria: A Machine Learning Approach to Uncover Key Risk Factors

**DOI:** 10.1002/puh2.70135

**Published:** 2025-09-29

**Authors:** Ibrahim Khalil Ja'afar, Olalekan A. Uthman

**Affiliations:** ^1^ Warwick Applied Health Warwick Centre for Global Health Warwick Medical School University of Warwick Coventry UK; ^2^ Department of Community Medicine University of Maiduguri Teaching Hospital Maiduguri Borno Nigeria

**Keywords:** childhood anaemia, demographic bias, machine learning, Nigeria, predictive modelling, risk factors

## Abstract

**Background:**

Childhood anaemia is a major public health challenge in Nigeria, with high prevalence among children under five. This study identifies key determinants and develops a predictive model using advanced machine learning technique.

**Methods:**

A total of 13,136 children aged 6–59 months from the 2018 National Demographic and Health Survey (NDHS) were analysed. Sixteen machine learning algorithms were evaluated on the basis of their ability to predict childhood anaemia using a wide range of individual, community and environmental factors. The Extra Trees (ET) classifier, demonstrating the highest predictive performance, was used to identify the top 10 predictors of childhood anaemia. A fairness and demographic bias assessment framework was incorporated to evaluate the model's performance across different regions, wealth index categories, ethnic groups and gender.

**Results:**

The ET classifier achieved an area under the curve (AUC) of 0.8319, an accuracy of 0.7565 and a recall of 0.7565. The top 10 predictors identified by the model included the number of under‐five children in the household, birth order, child age, media access, maternal health‐seeking behaviour, child gender, proximity to water, money problems, day land surface temperature and all population count. The demographic bias assessment revealed variations in model performance across different subgroups, with the lowest AUCs observed in the north‐east region (0.79), the poorest wealth index category (0.80) and the Hausa/Fulani ethnic group (0.81).

**Conclusion:**

This study shows that machine learning can accurately predict childhood anaemia in Nigeria and identify key risk factors, supporting targeted interventions. Future work should focus on refining models and integrating AI‐based interventions to reduce anaemia.

## Graphical abstract

Childhood anaemia affects about 60% of Nigerian children aged 6–59 months. Using nationally representative data, we applied 16 machine learning models to identify key predictors. The Extra Trees classifier performed best (*F*1 = 0.75), highlighting the potential of predictive modelling to guide targeted interventions and reduce childhood anaemia burden.

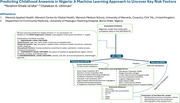

## 1 Introduction

Childhood anaemia remains one of the most pressing global health challenges, affecting approximately 269.7 million children under 5 years worldwide, with the highest burden concentrated in low‐ and middle‐income countries (LMICs) [1, 2]. Sub‐Saharan Africa bears a disproportionate burden, with prevalence rates exceeding 60% in many countries, making it a critical public health priority requiring urgent intervention [3, 4]. Nigeria, as Africa's most populous nation, faces particularly acute challenges, with childhood anaemia prevalence among children aged 6–59 months reaching alarming levels of approximately 68% according to recent national surveys [5, 6].

The adverse effects of childhood anaemia extend far beyond immediate health concerns, creating cascading impacts on child development, educational outcomes and long‐term economic productivity. Physiologically, anaemia impairs oxygen transport capacity, leading to reduced cellular metabolism and compromised organ function [7]. In the developing brain, iron deficiency anaemia during critical periods can cause irreversible damage to neural development, affecting cognitive function, memory formation and learning capacity [8, 9]. Research has consistently demonstrated that anaemic children exhibit significantly impaired cognitive development, including reduced attention span, decreased problem‐solving abilities and poor academic performance [10, 11]. A longitudinal study by Lozoff et al. [12] found that children with iron deficiency anaemia in infancy continued to show cognitive deficits and behavioural problems even after iron treatment, suggesting that early anaemia may cause permanent developmental damage. Similarly, motor development is significantly affected, with anaemic children showing delayed gross and fine motor skill development, reduced physical activity levels and impaired coordination [13, 14].

The immunological consequences of anaemia are equally concerning, as iron deficiency compromises immune system function, increasing susceptibility to infectious diseases and prolonging recovery periods [15, 16]. This creates a vicious cycle where anaemic children experience more frequent and severe infections, which further exacerbate nutritional deficiencies and anaemia severity [17]. Additionally, anaemia has been associated with increased mortality risk among children under five, particularly when combined with other nutritional deficiencies or infectious diseases [18, 19]. Long‐term economic implications are substantial, as childhood anaemia contributes to reduced educational attainment, lower adult productivity and decreased earning capacity [20, 21]. The World Bank estimates that iron deficiency anaemia costs developing countries 0.5%–2% of their gross domestic product through reduced productivity and increased healthcare expenditure [22].

Traditional approaches to anaemia prediction and risk assessment have relied primarily on conventional statistical methods, including logistic regression (LR) and survival analysis, which often fail to capture complex, non‐linear relationships among multiple risk factors [23, 24]. The emergence of machine learning (ML) techniques has revolutionized disease prediction and risk stratification, offering sophisticated approaches to analyse large, multidimensional datasets and identify subtle patterns that conventional methods might miss [25, 26]. Pioneering work in applying ML to childhood anaemia prediction began with Khan et al. [27], who conducted the first comprehensive study using data from the 2011 Bangladesh Demographic and Health Survey. Their analysis of 2013 children employed six ML algorithms, including random forest (RF), support vector machines (SVMs), *k*‐nearest neighbours (*k*‐NN) and classification and regression trees (CART), alongside traditional LR. The RF algorithm achieved the highest performance with 68.53% accuracy, 70.73% sensitivity, 66.41% specificity and an area under the curve (AUC) of 0.6857, demonstrating the potential of ML approaches for anaemia prediction [27].

Building on this foundation, subsequent research has expanded both methodologically and geographically. Tesfaye et al. [28] analysed 8482 children from the 2016 Ethiopian Demographic and Health Survey, comparing four ML algorithms with LR. Their study achieved improved performance metrics, with LR emerging as the best model (66% accuracy, 82% sensitivity, AUC 0.69), whereas RF achieved 64% accuracy and 0.63 AUC; notably, they highlighted the importance of data preprocessing and feature selection in optimizing model performance [28]. More recent advances have demonstrated even higher predictive accuracy through sophisticated methodological approaches. Using the 2016 Ethiopian DHS data with 9501 children, Yimer et al. reported an optimized RF model reaching 81.16% accuracy, 83.07% sensitivity, 79.26% specificity and AUC 0.818 [29].

Despite these methodological advances, several critical gaps remain in the literature. First, most existing studies have been limited to specific geographic contexts, primarily South Asian and East African settings, with limited representation from West African populations where anaemia burden is highest [29, 30]. Nigeria, despite having the largest population of children in Africa and among the highest anaemia prevalence rates globally, has been under‐represented in ML‐based anaemia prediction research. Second, although previous studies have achieved reasonable predictive accuracy, few have systematically addressed issues of algorithmic bias and demographic fairness. This is particularly important in diverse populations like Nigeria, where ethnic, regional and socio‐economic disparities may affect model performance across different subgroups [30]. Ensuring equitable prediction accuracy across demographic groups is crucial for developing inclusive health interventions. Third, most existing research has focused primarily on traditional socio‐demographic and health‐related predictors, with limited incorporation of environmental and climatic factors that may significantly influence anaemia risk. Given the established relationships between environmental conditions, infectious disease transmission and nutritional status, integrating environmental variables could enhance predictive accuracy and provide new insights for intervention strategies [31]. Fourth, although previous studies have compared multiple algorithms, the systematic evaluation of fairness and bias across demographic subgroups has been limited. This represents a critical gap, as deploying biased algorithms in healthcare settings could exacerbate existing health disparities rather than reduce them [26, 32].

To address these gaps, this study aims to develop and validate a comprehensive ML framework for predicting childhood anaemia in Nigeria using data from the 2018 National Demographic and Health Survey (NDHS). Our specific objectives are to: (i) systematically evaluate 16 ML algorithms to identify the optimal predictive model for childhood anaemia; (ii) incorporate comprehensive individual, household, community and environmental predictors to develop a holistic prediction framework; (iii) implement rigorous fairness and demographic bias assessment to ensure equitable model performance across different population subgroups; and (iv) identify key risk factors that can inform targeted intervention strategies. Given the complex interplay of socio‐economic, environmental, maternal and child‐related factors influencing childhood anaemia in Nigeria, and the demonstrated superiority of ML approaches in handling multidimensional health data, this study was designed to test specific hypotheses regarding the predictive capacity of these factors. We hypothesized that a combination of individual‐level factors such as child age, gender and birth order; household‐level factors, including wealth index, media access and number of under‐five children; maternal‐level factors such as education and health‐seeking behaviour; and environmental factors, including land surface temperature and proximity to water, can accurately predict childhood anaemia in Nigerian children aged 6–59 months using ML algorithms. Additionally, we hypothesized that advanced ensemble ML algorithms would demonstrate superior predictive performance compared to traditional LR models for childhood anaemia prediction.

To address these hypotheses, this study sought to answer three key research questions. First, which ML algorithm provides the highest predictive accuracy for childhood anaemia among Nigerian children? Second, what are the most important risk factors for childhood anaemia when analysed through ML approaches? Third, does model performance vary across different demographic subgroups, including regions, wealth categories, ethnic groups and gender? By addressing these questions, this research aims to bridge existing knowledge gaps and provide evidence‐based insights for developing targeted interventions to reduce the burden of childhood anaemia in Nigeria.

## 2 Method

### 2.1 Study Setting

Nigeria, spanning about 923,768 square kilometres, is Africa's most populous country [5]. In 2023, the World Bank estimated Nigeria's population at over 223 million [33]. The country is ethnically diverse, with 374 ethnic groups, with the Hausa/Fulani, Yoruba and Igbo being the largest. About 68% of the population lives in rural areas [5]. Administratively, Nigeria is divided into 37 states and 774 local government areas (LGAs) within 6 geopolitical zones: north‐east, north‐west, north‐central, south‐west, south‐south and south‐east.

Nigeria has three distinct tropical climate zones: monsoon climate in the south, savannah in the central regions and hot, semi‐arid climate in the north. Rainfall varies greatly: around 2000 mm falls annually in the south and 500 mm in the north [34]. These climate differences impact agricultural practices, which are key to Nigeria's economy [34].

### 2.2 Study Design and Sampling Technique

This is a population‐based cross‐sectional study using data from the 2018 NDHS [5]. The survey collected household data of children and their parents. The sampling frame was based on the 2006 Population and Housing Census. During the Census, localities were divided into enumeration areas (EAs), which served as the primary sampling units (PSUs) for the survey [5].

The 2018 NDHS used a stratified two‐stage sampling method. First, Nigeria's 36 states and the Federal Capital Territory were divided into urban and rural areas, creating 74 sampling strata. From each stratum, 1400 EAs were selected on the basis of the size. In the second stage, 30 households were systematically selected from each EA, resulting in 41,668 households.

### 2.3 Data Collection

The NDHS gathered data through in‐person interviews, achieving a 99% response rate, using structured questionnaires covering topics on biodata, demographics, socio‐economic and biomarkers [5]. Households were listed using tablets, and no replacements were made to avoid selection bias. Blood samples for anaemia testing were collected via finger prick. Blood samples for anaemia testing in children under five were collected using a finger prick (or heel prick for children aged 6–11 months). A drop of blood was drawn into a microcuvette and analysed on‐site with a portable, battery‐operated HemoCue analyser.

This study utilized data from the children's record (KR) file of the 2018 NDHS, with specific inclusion and exclusion criteria established to ensure data quality and relevance to the research objectives. Children were included in the analysis if they were aged 6–59 months at the time of survey, had undergone haemoglobin (Hb) testing during the survey with complete Hb measurements recorded, and whose mothers provided complete demographic and socio‐economic information from households that completed the full NDHS questionnaire. Conversely, children were excluded if they were under 6 months of age, as WHO anaemia thresholds are not applicable to this age group, or if they were aged 60 months and above. Additionally, children with missing or invalid Hb measurements, incomplete outcome data, or from households with incomplete survey responses for key variables were excluded from the analysis.

### 2.4 Data Preprocessing and Quality Control

All data preprocessing and ML analyses were conducted using PyCaret, an open‐source, low‐code ML library in Python that automates many aspects of the ML workflow. The preprocessing pipeline included several critical steps to ensure data quality and model performance.

Missing data handling was implemented through PyCaret's automated imputation strategies. For continuous variables, missing values were imputed using mean imputation, whereas categorical variables utilized mode imputation. Categorical variables were encoded using one‐hot encoding to convert them into numerical format suitable for ML algorithms. This process created binary dummy variables for each category while avoiding the dummy variable trap. Continuous variables were standardized using *z*‐score normalization to ensure all features contributed equally to model training, which is particularly important for distance‐based algorithms.

The dataset was randomly split into training (70%) and testing (30%) sets using stratified sampling to maintain the proportion of anaemic and non‐anaemic cases in both subsets. Feature scaling and transformation were applied only to the training set to prevent data leakage, with the same transformations subsequently applied to the test set.

### 2.5 Recruitment of Eligible Participants

This study analysed data on children aged 6–59 months from the 2018 NDHS. Of the approximately 14,000 eligible children who underwent anaemia testing across 42,000 surveyed households, only those with complete Hb data were included. After excluding 864 children with missing Hb measurements, a total of 13,136 children were retained for analysis.

### 2.6 Study Variables

#### 2.6.1 Outcome Variable

According to the WHO, children aged 6–59 months are considered anaemic if their Hb level is below 11.0 g/dL [35]. This study adopted similar guidelines. Anaemia status was recorded as a binary variable: 1 for anaemic and 0 for not anaemic.

#### 2.6.2 Explanatory Variables

Literature review informed the choice of variables for analysis, confirming direct links between individual factors and anaemia [36, 37], as well as strong associations with dietary habits [38]. Environmental factors, such as land surface temperature (LST) and average enhanced vegetation index (EVI), are also linked indirectly to childhood anaemia [39]. However, there is limited research that comprehensively explores the combined effects of individual, community and environmental factors on anaemia, as this study does.

##### 2.6.2.1 Individual‐Level Factors

Variables included (i) child‐specific factors like age, gender, birth order and diet quality; (ii) mother‐specific factors like age, education, ethnicity, religion, health insurance, decision‐making power and head of household status; and (iii) household‐specific factors like livestock ownership, wealth, access to media, water and sanitation.

##### 2.6.2.2 Community‐Related Factors

These are place of residence (rural or urban area), geopolitical region, ethnic diversity, community poverty, illiteracy and unemployment rates.

##### 2.6.2.3 Environment‐Related Factors

These are average rainfall, diurnal temperature range, proximity to water and drought episodes. Because the variables are continuous, mean values served as the reference for the analysis.

### 2.7 Ethical Consideration

This study uses publicly available secondary datasets from the DHS Programme, Nigeria. No additional ethical approval is needed. However, permission to use the data for research purposes was requested and granted before the commencement of this study.

The DHS follows strict ethical standards to protect respondents’ privacy and confidentiality. The survey has been approved by the Ethics Committee of ICF Macro, USA, which is involved in its management, and the National Ethics Committee in the Ministry of Health in Nigeria. All participants gave informed consent before participating, ensuring that the data are ethically sourced and respect their rights by protecting the privacy of the DHS survey respondents.

### 2.8 Statistical Analysis

#### 2.8.1 Modelling Approaches/Multiple ML Approaches

The 16 ML algorithms employed in this study were categorized into two distinct types based on their underlying learning mechanisms (Box 1).

**BOX 1 Explaining the machine learning (ML) algorithms**

**Table:** 

Type of algorithms	List of the ML algorithms and justification for use
Ensembling machine learning algorithms	Random forest classifier (RF), AdaBoost classifier (ADA), Gradient Boosting classifier (GBC), Extra Trees classifier (ET), Extreme Gradient Boosting (XGBoost), Light Gradient Boosting Machine (LightGBM) and CatBoost classifier (CatBoost), belong to ensemble learning, which can be used to solve classification and regression, and when combined can result in strong learners with high prediction accuracy [40]. This can be done either by boosting as in the case of RF or by bagging, for example, XGBoost Ensemble learning consists of many base learners. Unlike traditional machine learning approaches in which only one learner is trained with a single learning algorithm, ensemble learning consists of many base learners [41]. The prediction performance of a single learner is only slightly better than random guessing, but an ensemble increases it by combining to form strong learners with high prediction accuracy [42]. RF, ET, ADA, GBC, XGBoost, LightGBM and CatBoost have the advantage of handling complex data and interactions effectively
Non‐ensembling machine learning algorithms	Non‐Ensembling ML Algorithms, by contrast, make predictions using a single model, such as *K* Neighbours classifier (KN) for capturing local patterns, Naive Bayes (NB) for its probabilistic approach and Decision Tree classifier (DT) for understanding decision rules. SVM with a linear kernel was used for its effectiveness on linear data, while the Ridge classifier helped prevent overfitting [43] RF and ETs were used to handle data complexity and feature importance, and AdaBoost and Gradient Boosting improved model performance by focusing on errors in previous models. Advanced techniques such as XGBoost, LightGBM and CatBoost ensured high performance and processed large data sets efficiently [43]

Ensembling ML algorithms combine multiple base learners to create stronger predictors through collective decision‐making. These algorithms reduce prediction errors by leveraging model diversity and include RF classifier, Extra Trees (ET) classifier, AdaBoost classifier (ADA), Gradient Boosting classifier (GBC), Extreme Gradient Boosting (XGBoost), Light Gradient Boosting Machine (LightGBM) and CatBoost classifier. Ensemble methods operate through two main strategies: (i) bagging (e.g., RF and ET), where multiple models are trained on different data subsets and results are averaged, and (ii) boosting (e.g., AdaBoost and XGBoost), where models are trained sequentially to correct previous models’ errors.

Non‐ensembling ML algorithms rely on single model architectures to learn patterns and make predictions independently. These include *k*‐NN, Naive Bayes (NB), Decision Tree classifier (DT), SVM, Ridge classifier, LR, Linear Discriminant Analysis (LDA), Quadratic Discriminant Analysis (QDA) and Gaussian Process classifier (GPC). Each algorithm employs distinct mathematical frameworks—*k*‐NN captures local patterns through proximity‐based classification, NB uses probabilistic approaches based on Bayes’ theorem, DT creates interpretable decision rules, and SVM finds optimal separating hyperplanes.

Ensemble methods were included for their superior handling of complex feature interactions and robustness to data noise, particularly important given the multifactorial nature of childhood anaemia risk factors. Non‐ensemble algorithms served as benchmarks and provided interpretable alternatives, ensuring comprehensive evaluation across different learning paradigms. This dual approach enabled identification of the optimal predictive model while providing insights into algorithmic performance variations for childhood anaemia prediction.

##### 2.8.1.1 Hyperparameters Tuning, Model Development and Algorithm Comparison

Hyperparameter tuning: To achieve optimal performance from the ML models, hyperparameter tuning was conducted for each algorithm. The tuning process was carried out using a 10‐fold cross‐validation technique to ensure reliable and unbiased model evaluation.

Model development: Data preprocessing included handling missing values by replacing missing values with their mean, encoding categorical variables and splitting data into 70% and 30% for training and test sets, respectively. Each model was trained using the optimized hyperparameters to learn the anaemia risk factors.

**FIGURE 1:**
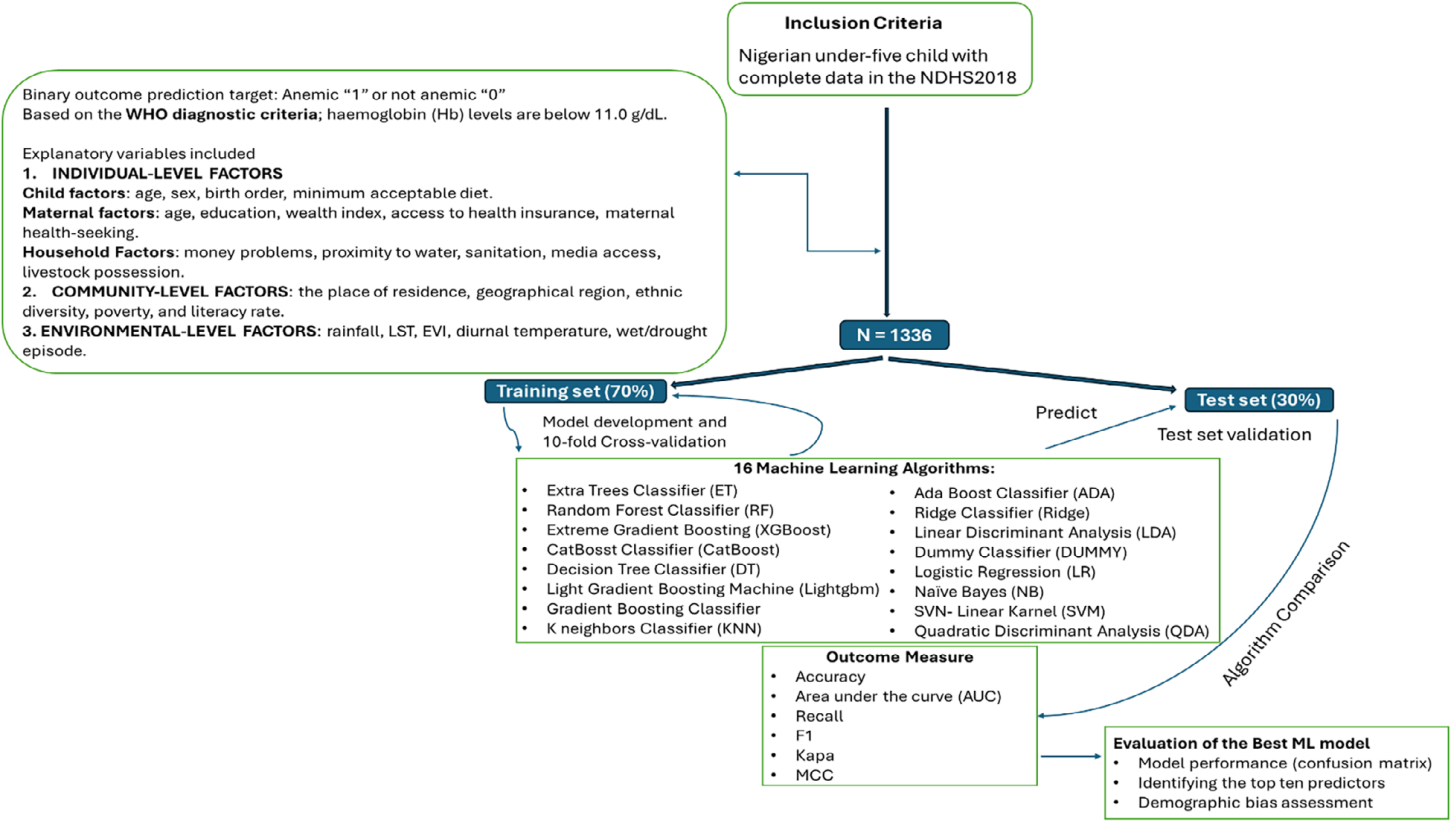
Steps followed in machine learning analysis.

Algorithm comparison and variable ranking: All the algorithms were compared to select the best. Their performance was assessed using metrics sensitive to imbalance, including precision, AUC, recall, Kappa, Matthews correlation coefficient (MCC), TT and *F*1‐score, in addition to overall accuracy. These measures provide a more balanced view of how well the models performed across both anaemic and non‐anaemic classes. *K*‐fold cross‐validation ensured reliability and generalizability. To gain a deeper understanding of the risk of childhood anaemia, the impact of all the variables was ranked on the basis of AUCs. These steps are summarized in Figure 1.

##### 2.8.1.2 Statistical Analysis

Pair‐wise corrected resampled *t*‐test was used for comparison of the predictive models’ performance of the ML algorithms in Python 3.6. Due to the multiple comparison problem, the significance level was adjusted to 0.008 using Bonferroni correction. A fairness and demographic bias assessment framework was incorporated to evaluate the model's performance.

## 3 Result

### 3.1 Sample Characteristics

The final analytical sample comprised 13,136 children aged 6–59 months, with 7799 (59.4%) classified as anaemic and 5337 (40.6%) as non‐anaemic (Table 1). This distribution indicates a relatively balanced outcome variable with no severe class imbalance that would necessitate synthetic balancing techniques. The prevalence of anaemia was consistent with national estimates, confirming the representativeness of our sample.

**TABLE 1:** Characteristics of the respondents.

		Overall	Anaemic	Not anaemic	*p* value
*N*		13,136	7799	5337	
Anaemic, *n* (%)	Anaemic	7799 (59.4)	7799 (100.0)	0 (0.0)	<0.001
	Not anaemic	5337 (40.6)	0 (0.0)	5337 (100.0)	
Mother age, *n* (%)	13–24	2865 (21.8)	1761 (22.6)	1104 (20.7)	0.024
	25–34	6750 (51.4)	3989 (51.1)	2761 (51.7)	
	35–49	3521 (26.8)	2049 (26.3)	1472 (27.6)	
Mother education, *n* (%)	No_formal education	5206 (39.6)	3332 (42.7)	1874 (35.1)	<0.001
	Primary school	2195 (16.7)	1295 (16.6)	900 (16.9)	
	Secondary school or higher	5735 (43.7)	3172 (40.7)	2563 (48.0)	
Wealth index, *n* (%)	Poorest	2762 (21.0)	1826 (23.4)	936 (17.5)	<0.001
	Poorer	2715 (20.7)	1680 (21.5)	1035 (19.4)	
	Middle	2898 (22.1)	1762 (22.6)	1136 (21.3)	
	Richer	2686 (20.4)	1541 (19.8)	1145 (21.5)	
	Richest	2075 (15.8)	990 (12.7)	1085 (20.3)	
Place of residence, *n* (%)	Rural	8196 (62.4)	5119 (65.6)	3077 (57.7)	<0.001
	Urban	4940 (37.6)	2680 (34.4)	2260 (42.3)	
Media access, *n* (%)	0	4654 (35.4)	2987 (38.3)	1667 (31.2)	<0.001
	1	3234 (24.6)	1935 (24.8)	1299 (24.3)	
	2	3849 (29.3)	2101 (26.9)	1748 (32.8)	
	3	1399 (10.7)	776 (9.9)	623 (11.7)	
Sex of children, *n* (%)	Female	6458 (49.2)	3857 (49.5)	2601 (48.7)	0.428
	Male	6678 (50.8)	3942 (50.5)	2736 (51.3)	
Children age, *n* (%)	0	65 (0.5)	33 (0.4)	32 (0.6)	0.398
	1	2546 (19.4)	1507 (19.3)	1039 (19.5)	
	2	2610 (19.9)	1545 (19.8)	1065 (20.0)	
	3	2577 (19.6)	1558 (20.0)	1019 (19.1)	
	4	2611 (19.9)	1567 (20.1)	1044 (19.6)	
	5	2727 (20.8)	1589 (20.4)	1138 (21.3)	
Birth order, *n* (%)	1	2528 (19.2)	1482 (19.0)	1046 (19.6)	0.007
	2	2360 (18.0)	1350 (17.3)	1010 (18.9)	
	3	2047 (15.6)	1188 (15.2)	859 (16.1)	
	4	1757 (13.4)	1054 (13.5)	703 (13.2)	
	5	4444 (33.8)	2725 (34.9)	1719 (32.2)	
Number of under‐5 children, *n* (%)	0	479 (3.6)	292 (3.7)	187 (3.5)	0.010
	1	3549 (27.0)	2060 (26.4)	1489 (27.9)	
	2	5198 (39.6)	3047 (39.1)	2151 (40.3)	
	3	2506 (19.1)	1508 (19.3)	998 (18.7)	
	4	867 (6.6)	554 (7.1)	313 (5.9)	
	5	537 (4.1)	338 (4.3)	199 (3.7)	
Health insurance, *n* (%)	No	12,852 (97.8)	7674 (98.4)	5178 (97.0)	<0.001
	Yes	284 (2.2)	125 (1.6)	159 (3.0)	
Female headed household, *n* (%)	No	11,591 (88.2)	6890 (88.3)	4701 (88.1)	0.668
	Yes	1545 (11.8)	909 (11.7)	636 (11.9)	
Have money problem, *n* (%)	Big problem	6536 (49.8)	4052 (52.0)	2484 (46.5)	<0.001
	No problem	6600 (50.2)	3747 (48.0)	2853 (53.5)	
Religion, *n* (%)	Christian	5952 (45.3)	3432 (44.0)	2520 (47.2)	<0.001
	Islam	7073 (53.8)	4312 (55.3)	2761 (51.7)	
	Others	111 (0.8)	55 (0.7)	56 (1.0)	
Ethnicity, *n* (%)	Hausa/Fulani	4672 (35.6)	2912 (37.3)	1760 (33.0)	<0.001
	Igbo	2106 (16.0)	1377 (17.7)	729 (13.7)	
	None	18 (0.1)	5 (0.1)	13 (0.2)	
	Others	4723 (36.0)	2665 (34.2)	2058 (38.6)	
	Yoruba	1617 (12.3)	840 (10.8)	777 (14.6)	
Household unsafe water, *n* (%)	Improved	9078 (69.1)	5291 (67.8)	3787 (71.0)	<0.001
	Unimproved	4058 (30.9)	2508 (32.2)	1550 (29.0)	
Household poor sanitation, *n* (%)	Improved	9203 (70.1)	5351 (68.6)	3852 (72.2)	<0.001
	None	241 (1.8)	163 (2.1)	78 (1.5)	
	Unimproved	3692 (28.1)	2285 (29.3)	1407 (26.4)	
Household air pollution, *n* (%)	No	2425 (18.5)	1220 (15.6)	1205 (22.6)	<0.001
	None	242 (1.8)	161 (2.1)	81 (1.5)	
	Yes	10,469 (79.7)	6418 (82.3)	4051 (75.9)	
Maternal health seeking, *n* (%)	0	1434 (10.9)	960 (12.3)	474 (8.9)	<0.001
	1	605 (4.6)	373 (4.8)	232 (4.3)	
	2	4411 (33.6)	2724 (34.9)	1687 (31.6)	
	3	4558 (34.7)	2576 (33.0)	1982 (37.1)	
	4	2128 (16.2)	1166 (15.0)	962 (18.0)	
Minimum acceptable diet, *n* (%)	No	11,741 (89.4)	7023 (90.1)	4718 (88.4)	0.003
	Yes	1395 (10.6)	776 (9.9)	619 (11.6)	
Geographical region, *n* (%)	North‐central	2262 (17.2)	1284 (16.5)	978 (18.3)	<0.001
	North‐east	2446 (18.6)	1486 (19.1)	960 (18.0)	
	North‐west	3333 (25.4)	1985 (25.5)	1348 (25.3)	
	South‐east	1818 (13.8)	1217 (15.6)	601 (11.3)	
	South‐south	1432 (10.9)	875 (11.2)	557 (10.4)	
	South‐west	1845 (14.0)	952 (12.2)	893 (16.7)	
Community ethnic diversity, mean (SD)		20.5 (22.9)	19.8 (22.8)	21.6 (23.1)	<0.001
Community poverty rate, mean (SD)		0.2 (0.3)	0.2 (0.3)	0.2 (0.3)	<0.001
Community illiteracy rate, mean (SD)		0.4 (0.4)	0.4 (0.4)	0.4 (0.4)	<0.001
Community unemployment rate, mean (SD)		0.0 (0.0)	0.0 (0.0)	0.0 (0.0)	
All population count, mean (SD)		99,467.9 (159,520.1)	97,608.4 (151,996.1)	102,190.7 (169,915.4)	0.118
Annual precipitation, mean (SD)		93.4 (40.8)	93.7 (42.2)	93.0 (38.8)	0.330
Aridity, mean (SD)		24.7 (15.9)	24.8 (16.3)	24.5 (15.2)	0.336
Day land surface temperature, mean (SD)		32.1 (2.9)	32.2 (3.0)	32.1 (2.9)	0.324
Diurnal temperature range, mean (SD)		11.2 (1.9)	11.2 (2.0)	11.1 (1.9)	0.004
Proximity to water, mean (SD)		152,611.9 (127,728.6)	154,262.5 (129,788.0)	150,195.1 (124,624.9)	0.074
Drought episodes, mean (SD)		4.7 (2.4)	4.7 (2.3)	4.7 (2.4)	0.175
Enhanced vegetation index, mean (SD)		2966.7 (793.6)	2959.8 (797.1)	2976.8 (788.4)	0.231
Frost days, mean (SD)		0.0 (0.0)	0.0 (0.0)	0.0 (0.0)	
Livestock cattle, mean (SD)		16.6 (26.1)	17.6 (27.6)	15.3 (23.7)	<0.001
Livestock chickens, mean (SD)		324.4 (549.0)	337.7 (577.0)	305.0 (504.7)	<0.001
Livestock goats, mean (SD)		70.7 (114.4)	76.5 (124.2)	62.1 (97.7)	<0.001
Livestock pigs, mean (SD)		10.6 (32.6)	8.9 (27.6)	13.2 (38.6)	<0.001
Livestock sheep, mean (SD)		46.4 (74.3)	48.7 (73.7)	43.1 (75.0)	<0.001
Maximum temperature, mean (SD)		32.9 (1.5)	33.0 (1.4)	32.7 (1.4)	<0.001
Mean temperature, mean (SD)		27.2 (0.9)	27.3 (0.9)	27.1 (1.0)	<0.001
Night land surface temperature, mean (SD)		20.5 (1.3)	20.5 (1.2)	20.5 (1.4)	0.014
Rainfall, mean (SD)		1240.9 (524.0)	1240.1 (539.3)	1242.2 (500.9)	0.821
Wet days, mean (SD)		7.3 (2.8)	7.2 (2.9)	7.3 (2.8)	0.020

Significant differences between anaemic and non‐anaemic children were observed across multiple socio‐demographic characteristics (*p* < 0.001). Maternal education showed a clear gradient, with anaemia prevalence highest among children of mothers with no education (42.7% vs. 35.1% in non‐anaemic children) and lowest among those with secondary or higher education (40.7% vs. 48.0%). Similarly, wealth index demonstrated a strong inverse association with anaemia, with the highest prevalence in the poorest quintile (23.4% vs. 17.5%) and lowest in the richest quintile (12.7% vs. 20.3%).

Geographical disparities were evident, with rural children experiencing higher anaemia rates (65.6% vs. 57.7% in urban areas). Regional variations showed the highest anaemia burden in the south‐east (15.6% vs. 11.3%) and lowest in the south‐west (12.2% vs. 16.7%). Environmental and community‐level factors, including livestock ownership, temperature variables and community poverty rates, showed statistically significant associations with anaemia status (*p* < 0.001).

### 3.2 Performance of ML Algorithms

The results of the comprehensive analysis, presented in Table 2, show that 12 of the 16 algorithms significantly outperformed the traditional LR model, demonstrating the superior predictive power of advanced ML techniques (*p* < 0.001). ET classifier achieved the highest performance with an impressive AUC of 0.8319. This outstanding performance was closely followed by the RF classifier with an AUC of 0.7857 and then XGBoost algorithm, which achieved an AUC of 0.7253. The ET classifier not only outperformed the AUC but also showed the highest accuracy (0.7565), recall (0.7565), precision (0.7547), *F*1‐score (0.7532), kappa (0.4849) and MCC (0.4882). These metrics reflect the ET classifier's exceptional ability to correctly identify cases of childhood anaemia while minimizing false positives and false negatives.

**TABLE 2:** Performance of different machine learning algorithms on predicting the risk of childhood anaemia.

	Model	Accuracy	AUC	Recall	Precision	*F*1	Kappa	MCC	TT (s)
ET	Extra Trees classifier	0.7565	0.8319	0.7565	0.7547	0.7532	0.4849	0.4882	1.8320
RF	Random forest classifier	0.7303	0.7857	0.73303	0.7274	0.7261	0.4278	0.4314	2.3710
XGBoost	Extreme Gradient Boosting	0.6809	0.7253	0.6809	0.6761	0.6758	0.3228	0.3257	3.2360
CatBoost	CatBoost classifier	0.6809	0.7281	0.6805	0.6762	0.6666	0.3039	0.3161	11.5620
DT	Decision Tree classifier	0.6737	0.6330	0.6737	0.6747	0.6741	0.3255	0.3257	0.7460
LightGBM	Light Gradient Boosting Machine	0.6670	0.7097	0.6670	0.6606	0.6541	0.2776	0.2868	2.0450
GBC	Gradient Boosting classifier	0.6384	0.6642	0.6384	0.6307	0.6055	0.1875	0.2097	3.6950
*k*‐NN	*k*‐nearest neighbours classifier	0.6326	0.6586	0.6326	0.6271	0.6285	0.2248	0.2259	0.5190
Ada	AdaBoost classifier	0.6231	0.6326	0.6231	0.6098	0.5929	0.1584	0.1740	1.1650
Ridge	Ridge classifier	0.6161	0.6238	0.6161	0.6007	0.5801	0.1361	0.1531	0.3240
LDA	Linear Discriminant Analysis	0.6161	0.6239	0.6161	0.6007	0.5816	0.1379	0.1541	0.5130
Dummy	Dummy classifier	0.5937	0.5000	0.5937	0.3525	0.4223	0.0000	0.0000	0.3170
LR	Logistic regression	0.5898	0.5620	0.5898	0.5302	0.4600	0.0029	0.0086	2.0570
NB	Naive Bayes	0.5787	0.5590	0.5787	0.5672	0.5674	0.0988	0.1008	0.3950
SVM	SVN‐linear kernel	0.5460	0.4962	0.5460	0.4788	0.4203	−0.0019	0.0008	0.4290
QDA	Quadratic Discriminant Analysis	0.5081	0.5074	0.5081	0.5275	0.4899	0.0212	0.0211	0.4630

Abbreviations: AUC, area under the curve; MCC, Matthews correlation coefficient.

In contrast, the SVM‐linear and QDA algorithms showed the lowest predictive power with AUCs of 0.4962 and 0.5074, respectively. These results highlight the importance of selecting the most appropriate algorithm for the task at hand, as the choice of algorithm can significantly affect the accuracy and reliability of predictions.

**FIGURE 2:**
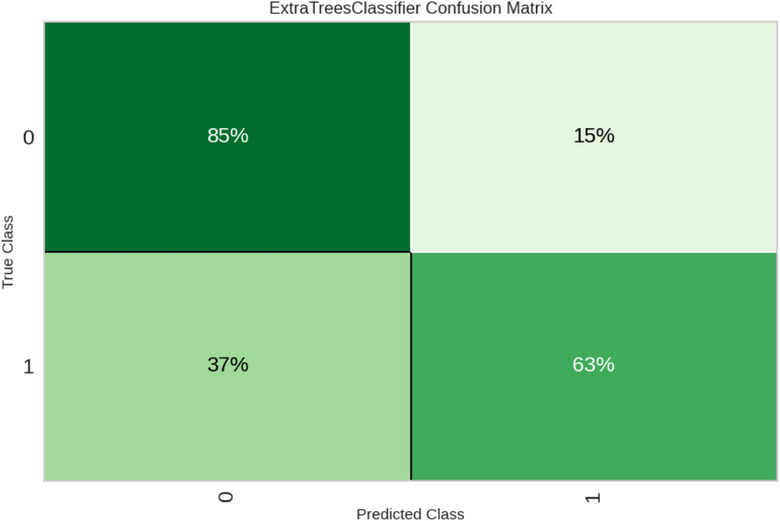
Confusion matrix for the best performing model—Extra Trees (ET) classifier.

To further evaluate the prediction performance of the ET classifier, a confusion matrix was constructed (Figure 2). This matrix provides a detailed breakdown of the model's ability to correctly classify anaemic and non‐anaemic cases, as well as the types of misclassifications that occur. The results show that the ET classifier correctly identified 85% of anaemic cases (true positive) and 63% of non‐anaemic cases (true negative). However, misclassification occurred: 15% of anaemic cases were incorrectly classified as non‐anaemic (false negative), and 37% of non‐anaemic cases were incorrectly classified as anaemic (false positive).

From a performance perspective, the presence of misclassifications highlights the importance of continually refining and improving the model. Future research could explore strategies to reduce false positives and false negatives, such as incorporating additional relevant features, optimizing model hyperparameters, or using ensemble methods that combine the strengths of multiple algorithms.

### 3.3 Ranking of Important Variables for Predicting Childhood Anaemia

**FIGURE 3:**
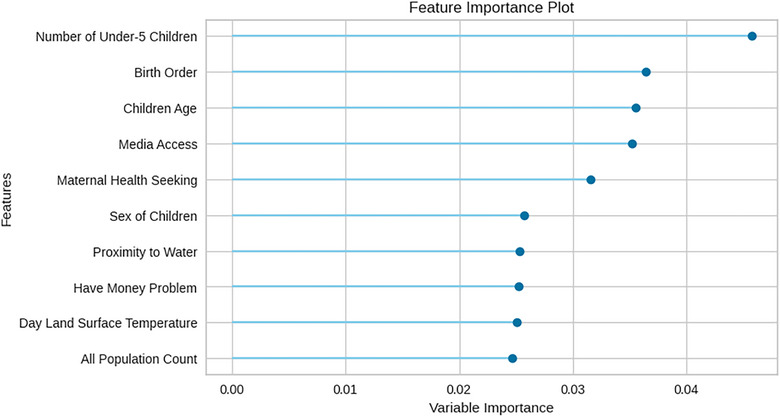
Ranking of important variables for predicting childhood anaemia.

Although identifying the best performing algorithm is critical, understanding the key factors driving anaemia in children in Nigeria is equally important for developing targeted interventions and public health strategies. Figure 3 shows variable ranking based on the mean decrease in AUC (expressed in percentage) for each characteristic and shows the relative importance of each factor in predicting childhood anaemia using the ET classifier.

### 3.4 Demographic Bias Assessment

**FIGURE 4:**
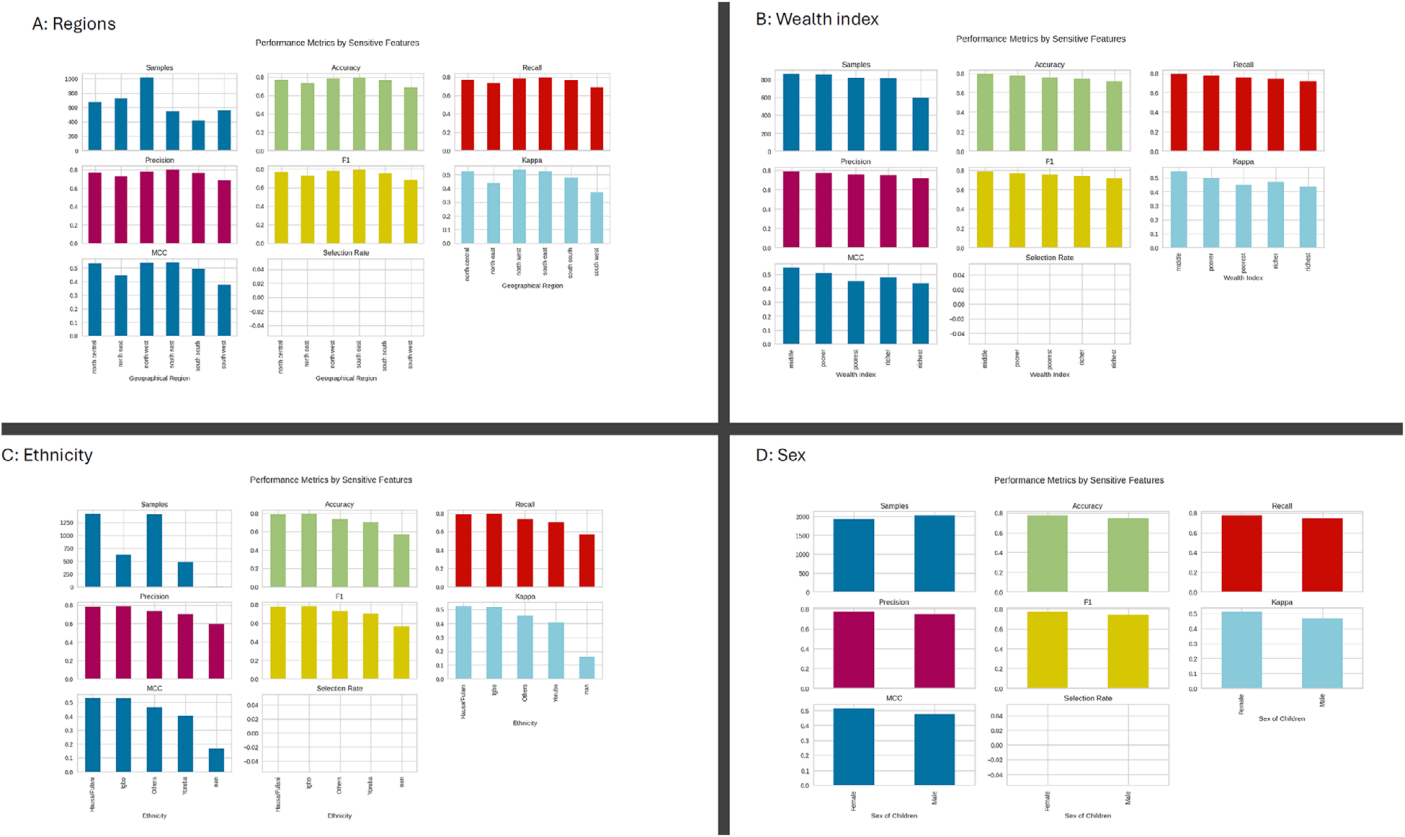
Demographic bias assessment results.

Central to the development of this predictive model is the incorporation of a fairness and demographic bias assessment framework.The results, as depicted in Figure 4, reveal some interesting patterns. This framework ensures the equitable application of the AI model across diverse populations, with initial stages of development integrating key demographic factors: geopolitical regions (A), Wealth index (B), Ethnicity (C), and sex (D) into the model as shown below.

Figure 4A shows the performance of the model across different regions of Nigeria. The model appears to perform best in the south‐south region, with an AUC of 0.85, followed by the north‐central region (AUC = 0.83). The lowest performance is observed in the north‐east region, with an AUC of 0.79. These regional variations may be attributed to differences in socio‐economic factors, healthcare access or environmental conditions that influence the prevalence and predictors of childhood anaemia.

Figure 4B presents the model's performance across different wealth index categories. The model demonstrates the highest AUC (0.86) for the richest wealth index category, whereas the lowest AUC (0.80) is observed for the poorest category. This finding suggests that the model may be more accurate in predicting childhood anaemia among children from wealthier households, possibly due to differences in nutritional status, healthcare utilization or other factors associated with socio‐economic status.

The model's performance across different ethnic groups is shown in Figure 4C. The highest AUC (0.84) is observed for the Igbo ethnic group, whereas the lowest AUC (0.81) is seen for the Hausa/Fulani group. These variations in model performance may reflect differences in cultural practices, dietary habits or genetic factors that influence the risk of childhood anaemia among different ethnic groups.

Finally, Figure 4D illustrates the model's performance based on the sex of the child. The model appears to perform slightly better for male children (AUC = 0.83) compared to female children (AUC = 0.82). Although this difference is relatively small, it warrants further investigation to ensure that the model does not perpetuate or amplify gender‐based disparities in anaemia risk prediction.

## 4 Discussion

### 4.1 Main Findings

This study leveraged advanced ML techniques to identify the key determinants of childhood anaemia in Nigeria and developed a predictive model with high accuracy and generalizability. The ET classifier emerged as the best‐performing model, achieving an AUC of 0.8319, an accuracy of 0.7565 and a recall of 0.7565, significantly outperforming traditional LR and other ML algorithms. The model identified the top ten predictors of childhood anaemia, which include household‐level factors such as the number of under‐five children and birth order, individual‐level factors such as child age and gender, maternal health‐seeking behaviour and environmental factors such as proximity to water and land surface temperature. These findings underscore the multifaceted nature of childhood anaemia, highlighting the interplay between individual, household and environmental determinants. Importantly, the study revealed variations in model performance across different demographic groups, with lower predictive accuracy observed in the north‐east region, the poorest wealth index category and the Hausa/Fulani ethnic group. These disparities suggest that socio‐economic, cultural and environmental factors significantly influence the risk of childhood anaemia and must be considered in the design of targeted interventions. The successful application of ML in this study demonstrates its potential to enhance our understanding of complex public health challenges and provides a robust framework for developing data‐driven solutions to reduce the burden of childhood anaemia in Nigeria and beyond.

### 4.2 Comparison With Other Studies

Our study's performance metrics align well with the existing literature, though some variations in performance metrics exist across different studies and contexts. The consistent identification of similar risk factors across studies (such as maternal education, wealth index, child age and geographic region) reinforces the validity of our findings and suggests that these factors are indeed fundamental predictors of childhood anaemia across different populations.

Several studies have applied ML algorithms to predict childhood anaemia with varying degrees of success. Khan et al. [27] conducted a pioneering study in Bangladesh using the 2011 BDHS data with 2013 children, achieving their best results with RF algorithm at 68.53% accuracy, 70.73% sensitivity, 66.41% specificity and AUC of 0.6857. Their LR achieved 62.75% accuracy with 63.41% sensitivity, 62.11% specificity and AUC of 0.6276. Notably, their study found *k*‐NN to have the poorest performance among all tested algorithms. In contrast, Tesfaye et al. [28] using the 2016 Ethiopian DHS data with 8482 children achieved higher performance metrics, with LR as their best model showing 66% accuracy, 82% sensitivity, 42% specificity and AUC of 69%. Their RF achieved 64% accuracy, 79% sensitivity, 42% specificity and AUC of 63%. The DT performed poorest with 60% accuracy in their study.

More recent studies have shown enhanced performance through advanced methodological approaches. Yimer et al. [29] achieved the highest reported performance using the 2016 Ethiopian DHS data with 9501 children, where RF achieved 81.16% accuracy, 83.07% sensitivity, 79.26% specificity and AUC of 81.80%. Their *k*‐NN model achieved 69.96% accuracy, whereas LR achieved 54.79% accuracy. This superior performance may be attributed to their comprehensive data preprocessing, SMOTE (Synthetic Minority Oversampling Technique) balancing technique and grid search hyperparameter optimization. Kebede Kassaw et al. [44] used the Boruta algorithm for feature selection with Ethiopian DHS 2016 data and identified key predictors, including number of children, distance to health facilities and health insurance coverage, though specific performance metrics were not extensively reported in their feature importance analysis. Zemariam et al. [45] focused on youth girls (aged 15–24) using 5642 samples from Ethiopian DHS 2016 data and achieved notable results with RF showing 82% AUC value, outperforming other algorithms including SVM (73.6% AUC), NB (66.3% AUC) and LR (67% AUC).

Several methodological and contextual factors contribute to the observed differences in performance across studies: Studies varied considerably in sample sizes, from 2013 children [27] to 9501 children [29]. Larger datasets generally provide better training opportunities for ML algorithms, potentially explaining some performance improvements in recent studies. Studies conducted in different countries (Bangladesh vs. Ethiopia) represent different populations with varying anaemia prevalence rates, risk factor distributions and healthcare contexts. The prevalence of anaemia varied from approximately 40% in Ethiopian studies to higher rates in the Bangladesh study, which can impact model performance and generalizability. The implementation of advanced data preprocessing techniques, particularly data balancing methods, significantly influenced performance. Yimer et al. [29] employed SMOTE which demonstrated superior performance compared to unbalanced data approaches used in earlier studies. Similarly, the application of sophisticated hyperparameter tuning through grid search optimization contributed to enhanced model performance. Studies employed different feature selection approaches. Although earlier studies relied on traditional statistical significance testing, more recent studies utilized advanced algorithms like Boruta [44, 45] for more sophisticated feature selection, potentially improving model accuracy. Variations in algorithm implementation, including different software packages, hyperparameter settings and validation techniques, contributed to performance differences. Studies using more recent implementations with optimized parameters generally achieved better results.

The observed variations in performance metrics across studies highlight the importance of methodological considerations in ML applications for health prediction.

Our study contributes to this growing body of evidence by providing a robust predictive framework using the ET classifier (AUC = 0.8319) on a large Nigerian dataset, incorporating innovative demographic bias assessment to evaluate model fairness across different population subgroups and identifying novel environmental predictors such as day land surface temperature alongside traditional socio‐demographic factors, while acknowledging that continued methodological refinements and larger datasets may further enhance predictive accuracy in future research.

### 4.3 Implications for Policy and Future Research

The findings of this study have significant implications for policy and future research aimed at reducing the burden of childhood anaemia in Nigeria. Although individual‐level factors are important, the study highlights the significance of household‐level factors (e.g., number of under‐five children and media access) and maternal factors (e.g., health‐seeking behaviour). This suggests that interventions should not only focus on the child but also consider the broader family and community context. Future research could explore the effectiveness of integrated interventions that simultaneously address multiple risk factors at different levels.

Policymakers should focus on improving mothers’ education and health knowledge because these factors have been shown to positively affect children's health in an Indian study [46]. Interventions that promote family planning and birth spacing could also help reduce the number of young children in households, thereby alleviating the resource constraints that may contribute to increased anaemia risk. Additionally, using media to educate about anaemia prevention could be a powerful way to spread important health information and improve child health outcomes.

Moreover, the study's findings on demographic bias in model performance have important implications for ensuring equitable healthcare delivery. The variations in model performance across regions, wealth index categories, ethnic groups and sex highlight the need for tailored approaches that consider the unique socio‐economic, cultural and environmental factors influencing childhood anaemia in different subpopulations. Policymakers should prioritize efforts to reduce disparities in healthcare access and quality across these demographic groups. This may involve targeted resource allocation, culturally sensitive interventions and the engagement of local communities in the design and implementation of anaemia prevention and control programmes.

Furthermore, the successful application of ML techniques in this study highlights the potential for AI‐based approaches to revolutionize anaemia risk prediction and management. Future research should explore the integration of these predictive models into clinical decision support systems and mobile health applications. By providing healthcare workers with real‐time risk assessments and personalized recommendations, these tools could greatly enhance the early detection and timely management of childhood anaemia.

However, the implementation of AI‐based interventions in low‐resource settings may face challenges related to infrastructure, technical expertise and cultural acceptability. Future research should therefore focus on developing strategies for the ethical and responsible deployment of these technologies, ensuring that they are accessible, affordable and aligned with local needs and values.

### 4.4 Strengths and Limitations of the Study

This study presents several methodological and analytical strengths that enhance the reliability and validity of our findings. First, we utilized a large, nationally representative dataset from the 2018 Nigerian Demographic and Health Survey comprising 13,136 children aged 6–59 months, providing substantial statistical power and ensuring findings are generalizable to the Nigerian population. The comprehensive nature of the NDHS data allowed for the inclusion of diverse predictors spanning individual, household, community and environmental factors, enabling a holistic understanding of childhood anaemia determinants. Our methodological approach demonstrates several strengths in ML application. We implement mted and compared 16 different ML algorithms, providing a robust evaluation framework to identify the optimal predictive model. The systematic application of advanced data preprocessing techniques, including comprehensive feature selection and data balancing methods, ensured optimal model performance. The incorporation of rigorous cross‐validation procedures and hyperparameter optimization through grid search enhanced model reliability and prevented overfitting.

A notable strength of this study is the integration of fairness and demographic bias assessment, which represents an innovative approach in health prediction modelling. By evaluating model performance across different regions, wealth categories, ethnic groups and genders, we ensured equitable prediction capabilities and identified potential disparities in model accuracy across population subgroups. This approach addresses critical concerns about algorithmic bias in healthcare applications and promotes health equity considerations. The study's analytical rigour is further strengthened by the identification of novel environmental predictors, including day land surface temperature and proximity to water sources, which expands the traditional understanding of anaemia risk factors beyond conventional socio‐demographic variables. The use of geospatial and environmental data provides new insights into the complex interplay between environmental conditions and childhood anaemia risk.

Despite these strengths, several limitations must be acknowledged. The cross‐sectional nature of the NDHS data limits our ability to establish causal relationships between identified predictors and childhood anaemia. Although we can identify associations and develop predictive models, temporal relationships and causality cannot be definitively established, requiring cautious interpretation of findings for intervention design. The reliance on secondary data presents inherent constraints, as we were limited to variables available in the NDHS dataset. Important clinical variables such as detailed dietary intake, specific micronutrient deficiencies, parasitic infections and chronic disease status were not available, potentially limiting the comprehensiveness of our predictive model. Additionally, some variables relied on self‐reported information, which may introduce recall bias and measurement error.

The generalizability of our findings may be limited to the Nigerian context and similar sub‐Saharan African settings. Although the model demonstrated strong performance within the study population, external validation in different geographic contexts, healthcare systems or time periods would be necessary to confirm broader applicability. Cultural, socio‐economic and healthcare infrastructure differences across countries may affect model performance and predictor importance.

Technical limitations include the potential for algorithmic bias despite our fairness assessment efforts. Although we evaluated performance across major demographic subgroups, more granular analyses of minority populations or specific vulnerable groups may reveal additional disparities. The ML models, while achieving good predictive accuracy, remain ‘black box’ approaches with limited clinical interpretability for some algorithms, potentially hindering adoption in clinical practice. Data quality considerations include the potential for missing data patterns that may not be completely random, despite our imputation strategies. The standardized NDHS protocols, while ensuring consistency, may not capture local variations in anaemia determinants or cultural factors specific to different Nigerian regions. The study's focus on prediction rather than intervention limits its immediate clinical utility. Although the identified risk factors provide valuable insights, translating these findings into actionable intervention strategies requires additional research on the effectiveness and feasibility of targeting specific predictors.

Finally, the temporal gap between data collection (2018) and analysis may limit the currency of findings, particularly given potential changes in healthcare policies, economic conditions and environmental factors that could affect anaemia prevalence and risk factor patterns. Future studies should consider more recent data and longitudinal designs to address these limitations and enhance the clinical utility of ML approaches for childhood anaemia prediction and prevention. Despite these limitations, this study provides valuable contributions to the understanding of childhood anaemia prediction using advanced analytical methods and establishes a foundation for future research incorporating more comprehensive clinical data and intervention studies.

## 5 Conclusion

This study highlights the utility of ML techniques in accurately predicting childhood anaemia in Nigeria while identifying key risk factors that could guide targeted public health interventions. After evaluating various ML algorithms, ET was chosen for superior predictive accuracy and AUC. Consequently, an ET‐based anaemia prediction model for Nigerian children was successfully developed, and its performance across different demographic groups was assessed to ensure equitable risk prediction. The model could be ready for implementation following external validation. Future research should prioritize refining this predictive model to improve its precision and applicability across diverse populations, as well as integrating it into broader health systems for real‐time monitoring and decision‐making.

Additionally, efforts should be directed towards designing and evaluating comprehensive, AI‐driven intervention strategies that address the immediate causes of childhood anaemia and the underlying socio‐economic and environmental determinants. Scaling these innovations across similar settings could significantly advance global efforts to reduce the burden of childhood anaemia and improve child health outcomes in resource‐constrained regions.

## Author Contributions

**Ibrahim Khalil Ja'afar**: conceptualization (lead), methodology, data curation, project administration, writing – original draft (lead), writing – review and editing (equal). **Olalekan A. Uthman**: conceptualization (supporting), formal analysis, writing – review and editing (equal), validation, supervision.

## Conflicts of Interest

The authors declare no conflicts of interest.

## Data Availability

The data used in this study are publicly available from the Demographic and Health Surveys (DHS) Programme. The 2018 Nigeria Demographic and Health Survey (NDHS) dataset can be accessed upon reasonable request from the DHS Programme website (https://submission.wiley.com/submission/submissionBoard/75025d2f‐9811‐490d‐91c1‐015c06f94ebe) after a brief registration and approval process.
